# Analytical validation of real-time quantitative PCR assays for optimum diagnosis of vivax malaria

**DOI:** 10.1590/0074-02760180350

**Published:** 2019-01-31

**Authors:** Natália Ketrin Almeida-de-Oliveira, Otacílio C Moreira, Aline Rosa de Lavigne, Leila Mendonça-Lima, Guilherme Loureiro Werneck, Cláudio Tadeu Daniel-Ribeiro, Maria de Fátima Ferreira-da-Cruz

**Affiliations:** 1 Fundação Oswaldo Cruz-Fiocruz, Instituto Oswaldo Cruz, Laboratório de Pesquisa em Malária, Rio de Janeiro, RJ, Brasil; 2 Fundação Oswaldo Cruz-Fiocruz, Instituto Oswaldo Cruz, Laboratório de Biologia Molecular e Doenças Endêmicas, Rio de Janeiro, RJ, Brasil; 3 Fundação Oswaldo Cruz-Fiocruz, Instituto Oswaldo Cruz, Laboratório de Genômica Funcional e Bioinformática, Rio de Janeiro, RJ, Brasil; 4 Universidade do Estado do Rio de Janeiro, Instituto de Medicina Social, Departamento de Epidemiologia, Rio de Janeiro, RJ, Brasil

**Keywords:** malaria, diagnosis, Plasmodium vivax

## Abstract

**BACKGROUND:**

The prompt diagnosis of plasmodial species for effective treatment prevents worsening of individual health and avoids transmission maintenance or even malaria reintroduction in areas where *Plasmodium* does not exist. Polymerase chain reaction (PCR) allows for the detection of parasites below the threshold of microscopic examination.

**OBJECTIVE:**

Our aim was to develop a real-time PCR test to reduce diagnostic errors and increase efficacy.

**METHODS:**

The lower limit of quantification and the linearity/analytical sensitivity to measure sensitivity or limit of detection (LoD) were determined. Intra-assay variations (repeatability) and alterations between assays, operators, and instruments (reproducibility) were also assessed to set precision.

**FINDINGS:**

The linearity in SYBR™ Green and TaqMan™ systems was 10^6^ and 10^2^ copies and analytical sensitivity 1.13 and 1.17 copies/μL, respectively. Real-time PCR was more sensitive than conventional PCR, showing a LoD of 0.01 parasite (p)/μL. Reproducibility and repeatability (precision) were 100% for up to 0.1 p/μL in SYBR™ Green and 1 p/μL in TaqMan™ and conventional PCR.

**CONCLUSION:**

Real-time PCR may replace conventional PCR in reference laboratories for *P. vivax* detection due to its rapidity. The TaqMan™ system is the most indicated when quantification assays are required. Performing tests in triplicate when diagnosing *Plasmodium*-infected-asymptomatic individuals is recommended to minimise diagnostic errors.

*Plasmodium vivax* is the most common malaria parasite outside Africa and is the predominant species in Latin America,^1^ causing 87% of malaria cases in Brazil.^2^ Currently, the number of malaria cases worldwide is decreasing due to significant advancements toward malaria elimination. However, *P. vivax* elimination represents a challenge larger than that of *Plasmodium falciparum* due to the presence of hypnozoites, the dormant form in the liver. Thus, clinical infection must be identified and treated as soon as it appears.^3^ Identification and treatment of asymptomatic individuals are also obligatory since these silent carriers harbour the parasite and perpetuate transmission within the community.^4^ Microscopy and rapid diagnostic tests are unreliable at low parasitaemia levels.^5^ ^,^ ^6^ ^,^ ^7^ In 2014, the World Health Organization (WHO) considered 2 p/μL as the lower detection limit for microscopy;^3^ however, the minimum number of p/µL that perpetuates transmission in low transmission settings remains uncertain. Submicroscopic *P. vivax* infections can be particularly prevalent in areas of low transmission, and ~70% can only be detected by polymerase chain reaction (PCR).^8^ Several PCR tests for malaria diagnosis have been developed, but their reported sensitivities vary widely,^8^ ^,^ ^9^ as well as the results between laboratories for the same patient sample.^8^ ^,^ ^9^ ^,^ ^10^ ^,^ ^11^ Indeed, PCR results require careful standardisation when the target population includes low-grade parasitaemic individuals.^11^ An in-house conventional PCR protocol for *P. vivax* detection previously standardised by our group has been effective for detecting infection,^12^ but conventional PCR is not as fast as real-time PCR and requires post-PCR handling, increasing the risk of contamination, labour time, and reagent costs.^13^ In addition, the workflow checklist for standardisation, including the essential parameters for validation of these methodologies, was not found using a PubMed *Molecular Malaria Diagnosis Reference Center* keyword search.

Thus, the aim of this study was to develop a real-time PCR assay for *P. vivax* malarial diagnosis and compare its performance with conventional PCR, including protocols to assess precision (repeatability and reproducibility), limit of detection, and analytical sensitivity.

## MATERIALS AND METHODS

*Samples* - A total of eight blood samples collected in Tucuruí Pará state, located in the Amazon Region of Brazil (S 3º 46’ W 49º 40’) from vivax malaria patients with different p/μL diagnosed by Giemsa-stained thick blood smears (sample 1: 5.280 p/μL, sample 2: 1.000 p/μL, sample 3: 17.320 p/μL, sample 4: 800 p/μL, sample 5: 32 p/μL, sample 6: 10.000 p/μL: sample 7: 100 p/μL, and sample 8: 120 p/μL) were used. Besides *P. vivax* PCRs,^12^ all samples were tested by PCR for *P. falciparum* ^14^ and *Plasmodium malariae*.^15^ Only monoinfected *P. vivax* patients were included in the study. Sample 7 (100 p/µL) was divided into five aliquots (from 1 to 5), and then each 200 µL aliquot was serially diluted in 1800 µL uninfected human blood from 10^2^ to 10^-7^ p/μL, generating 50 samples to analyse PCR parameters.

*PCR protocols* - Conventional PCR was performed using the cysteine proteinase gene (accession number PVX117565) as amplification target, as described elsewhere.^12^ For increasing amplification efficacy in quantitative PCR (qPCR), the size of the PCR product was reduced by designing new primers using Primer Express™ v3.0 (Applied Biosystems, CA, USA) and Oligo Analyzer™ 3.1 (Integrated DNA Technologies, Skokie, IL, USA). In this way, the forward primer Pv1 (5’-ATC AAC GAG CAG ATG GAG AAA TAT A-3’) was maintained while the reverse primer Pv6^12^ was replaced by Pv5 (5’-GCT CTC GAA ATC TTT CTT CGA-3’), resulting in an annealing temperature increased to 60ºC and the amplicon size reduced from 262 to 134 bp. The specificity of the new target was investigated using the NCBI BLAST sequence analysis database.

*DNA extraction efficiency* - DNA of all 58 blood samples (eight blood samples plus 50 diluted samples obtained from one of the eight samples with 100 p/mL) was extracted from 1 mL whole blood using a QIAamp™ DNA Blood Midi Kit (QIAGEN, Hilden, Germany), as described by the manufacturer. DNA was stored at -20ºC until used. β-globin gene amplification was carried out according to a protocol previously described^16^ as internal reference control in conventional PCR and qPCR SYBR™ Green experiments, to assess both DNA extraction failure and amplification inhibition.

The TaqMan™ assay consisted of a multiplex reaction to simultaneously detect *P. vivax* and an internal reference human DNA control, using a commercial TaqMan™ RNaseP Control 20× kit (Applied Biosystems). The RNaseP DNA internal reference was tested in three different concentrations: 0.1×, 0.5×, and 1×.

*qPCR standardisation* - For comparative purposes, qPCR assays were performed with SYBR™ Green and TaqMan™ probe systems. The first step of standardisation was to define the optimal Pv1 and Pv5 primer concentration without primer-dimer formation. Subsequently, three different concentrations (0.1, 0.2, and 0.3 µM) of each primer generating a matrix of nine different combinations were tested in both SYBR™ Green and TaqMan™ systems. The probe Pviv was designed (5’-FAM-AAC TTC AAA ATG AAT TAT CTC-MGB-NFQ-3’) for TaqMan™ assays, and was assessed at six different concentrations (0.05, 0.1, 0.15, 0.2, 0.25, and 0.3 µM). All assays for primers and probe standardisations were performed using sample 2 in duplicate. The SYBR™ Green and TaqMan™ reactions comprised 22.5 µL of mix containing 1× Master Mix, Pv1 and Pv5 primers, and UltraPure™ Distilled Water (Invitrogen, CA, USA) plus 2.5 µL template DNA. For the SYBR™ Green assay, Power SYBR™ Green Master Mix 2× (Applied Biosystems) was employed, while for TaqMan™, Universal Master Mix 2× (Applied Biosystems) and Pviv probe were used. For TaqMan™ a multiplexing assay using the RNaseP Detection Reagent Kit (Applied Biosystems) was performed. To analyse amplification interference between *P. vivax* primers and probes with RNaseP primers to guarantee amplification efficiency in a single assay tube, sample 8 was tested in two different concentrations: 120 and 0.12 p/µL.

The thermal conditions for both SYBR™ Green and TaqMan™ systems included an initial hold (95ºC for 10 min), followed by 40 cycles (95ºC for 15 s and 60ºC for 1 min). For SYBR™ Green reactions, after amplification the product, was subjected to melt curve analysis (95ºC for 15 s; 60ºC for 1 min; 95ºC for 30 s; 60ºC for 15 min). The reactions were run in 96-well optical plates in an Applied Biosystems 7500 Real-Time PCR System Apparatus and the results were analysed using 7500 Software v. 2.0.6.

All reactions included a positive control (DNA sample from a *P. vivax* infection diagnosed by microscopy examination), negative control (DNA sample extracted from blood donor human leucocytes), and non-template control (NTC; PCR reaction without template DNA).

*qPCR efficiency* - The efficiency (capacity to duplicate DNA during a PCR cycle), linearity (reportable range), and limit of quantification (LoQ) were assessed using a standard curve constructed with plasmid DNA, such that 1 parasite corresponded to 1 DNA copy. For this purpose, a 262 bp *P. vivax* DNA fragment of the cysteine proteinase gene with a single copy per haploid genome^12^ was cloned using a TOPO™ TA Cloning kit (Invitrogen). The cloned *P. vivax* DNA was serially diluted in 1× Tris-EDTA (TE) buffer from 4 × 10^6^ to 4 × 10^0^ copies/μL and each dilution point was tested in triplicate. The data analysis was performed using SigmaPlot™ v. 12.0 (Systat Software Inc) by linear regression considering the determination coefficient (r^2^), y-intercept, and slope values.

*Analytical sensitivity (LoD)* - Analytical sensitivity or LoD is the ability of the assay to detect very low parasite DNA concentrations and corresponds to the lowest concentration of the analyte (DNA) in a sample that can be consistently detected at a 95% confidence level.^17^ ^,^ ^18^ To calculate such sensitivity in qPCR, seven concentrations of the cloned *P. vivax* DNA, close to the lower limit of reportable range (LoQ), were serially diluted (1:2) in human DNA from an uninfected donor to reproduce interference of human DNA in low parasitaemia conditions. To generate robust results, each concentration was tested 12 times for five days, generating 60 assays per concentration, and the same lot of reagents was employed in all assays, as recommended.^17^ Data were exported to the statistical software Minitab 15™ (Minitab Inc., State College, PA, USA) and submitted to binomial regression analysis by the Probit statistical model defined by Napierian logarithm. In the case of qualitative PCRs (conventional and real-time formats), sample 7 was divided into five aliquots, and each was diluted from 10^2^ to 10^-7^ p/μL to establish the LoD. The same dilutions were tested three times a day during three consecutive days to investigate variability among the assays. LoD was the lowest concentration showing at least one positive replicate on each of the three days tested.

*Analytical specificity* - In addition to the BLAST evaluation, the Pv1/Pv5 primer set was also tested against DNA of other *Plasmodium* spp. that infect humans, including *P. falciparum*, *P. malariae*, and *Plasmodium ovale*, to investigate if the target detection was affected by cross-reactivity. Samples were tested in duplicate by real-time PCR.

*Precision* - To assess the repeatability and reproducibility, agreement between real-time and conventional PCR was considered. To this end, the same sample and protocol described for LoD were used.^17^ ^,^ ^18^ Repeatability was analysed among triplicates of an assay (within-run), whereas reproducibility was determined in three repeated day assays (between-runs), between operators in a single-day assay and between two GeneAmp™ 9700 PCR Systems and one Veriti™ fast Thermal Cycler (both from Applied Biosystems).

Kappa (κ) coefficient was utilised to measure agreements between operators and equipments not occurred by chance.^19^

*Ethics approval and consent to participate* - After obtaining informed consent, venous blood collection was performed according to protocols previously approved by the Ethical Research Committees of the Oswaldo Cruz Foundation (Fiocruz) (32839013.6.00005248).

## RESULTS

**Fig. 1:**
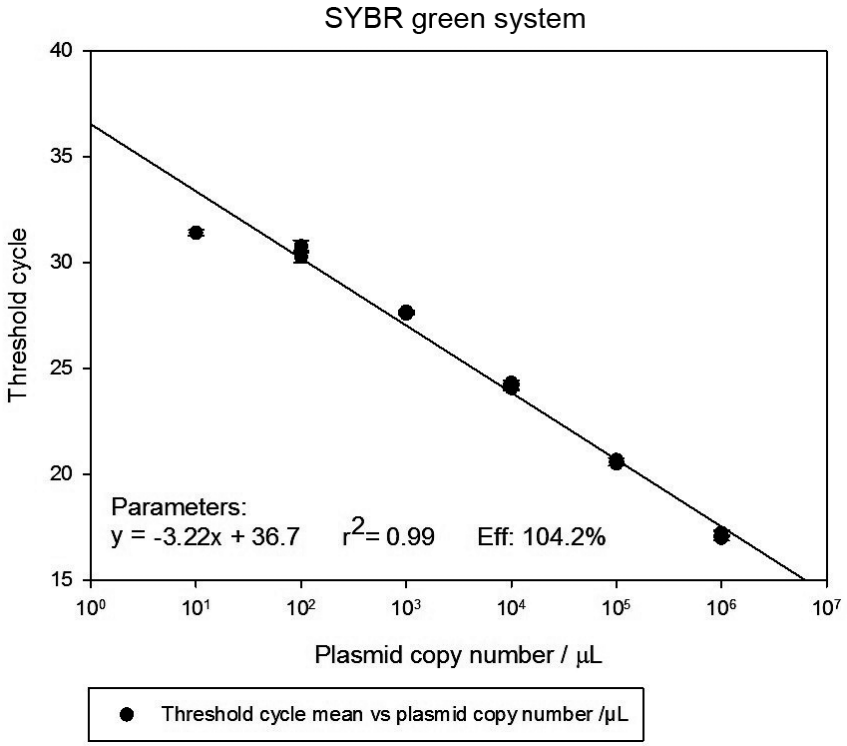
standard curve containing cloned DNA from 10^6^ to 10^1^ copies/µL, amplified in triplicate. Serial dilutions were prepared in triplicate. The x-axis plots Ct values and the y-axis plots log of initial input cloned DNA copy number. The linear range through r^2^ value of 0.999 for SYBR™ Green.

*qPCR standardisation* - Primer set Pv1/Pv5 was tested in duplicate in 9 combinations to select the concentration for each primer that generated the lowest cycle threshold (Ct) value. TaqMan™ showed the better performance at 0.3 µM for both primers, while SYBR™ Green varied according to the primer: 0.3 µM for Pv1 and 0.2 µM for Pv5. The melt curve analysis in SYBR™ Green system revealed a successful outcome with a single peak at ~72ºC, without evidence of primer-dimer formation [Supplementary data I (Tables I-II, Fig. 1)].

To confirm the specificity of the DNA target (134 bp), 5 µL of each primer concentration was tested by agarose gel electrophoresis, and no unspecific amplification was detected.

**Fig. 2:**
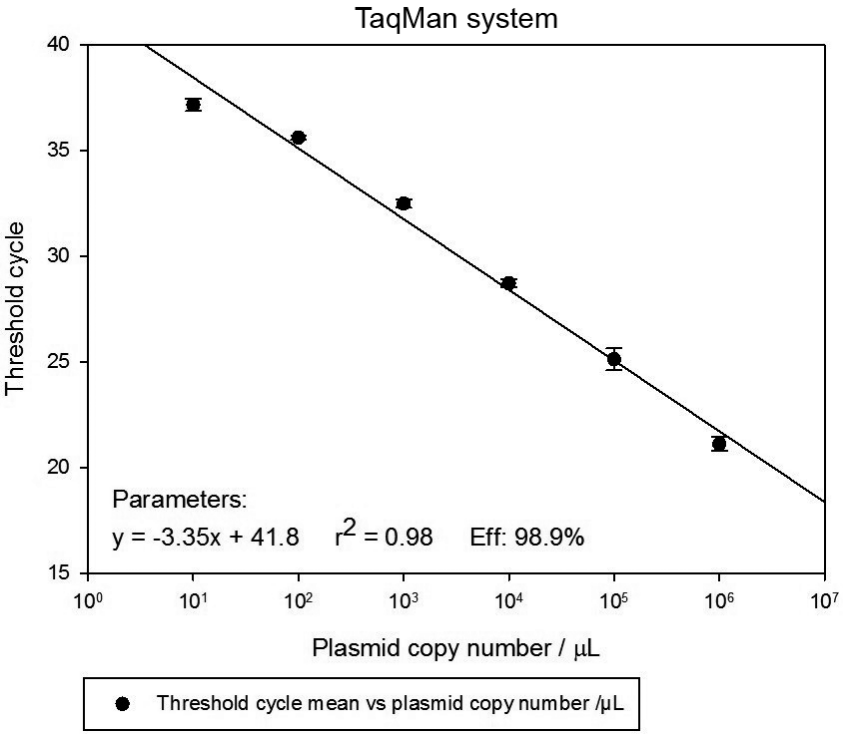
standard curve containing cloned DNA from 10^6^ to 10^1^ copies per µL; amplified in triplicate. Serial dilutions were prepared in triplicate. The x-axis plots Ct values and the y-axis plots log of initial input cloned DNA copy number. The linear range through r^2^ value of 0.98 for TaqMan™.

TaqMan™ probe and internal reference concentrations were also investigated. The probe concentration of 0.15 µM was the lowest concentration showing Ct values similar to those verified in higher concentrations; therefore, it was selected for TaqMan™ assays. No significant Ct differences between 0.5- and 1-fold concentration were detected when the internal reference gene RNaseP was tested; consequently, the 0.5-fold concentration was adopted to minimise cost [Supplementary data I (Tables III-IV, Fig. 2)]. With respect to the multiplexing assay, two positive controls were applied (0.12 and 120 p/µL) and calibration status showed similar amplifications when compared to singleplex reactions (Table I).

*qPCR efficiency* - After standardisation, PCR efficiency was determined by DNA serial dilutions to define reportable range and LoQ. The curve plot of 6-fold serial dilutions (10^6^ to 10^1^ copies/µL) displayed acceptable linear range for SYBR™ Green (r^2^ = 0.99) and TaqMan™ systems (r^2^ = 0.98), as well as similar amplification efficiencies for SYBR™ Green (104.2%) and TaqMan™ (98.9%). The linearity range of both assays was 10^6^ to 10^2^ copies/µL, generating a LoQ of 10^2^ copies/µL (Figs 1-2).

*Analytical sensitivity (LoD)* - The LoD defines the lower concentration that could be used as a positive control in PCR assays. Since analytical sensitivity most often resides below the linear range, a cloned cysteine proteinase gene was used to generate *P. vivax* DNA dilutions (4, 2, 1, 0.5, 0.25, 0.1, and 0.06 copies/µL) for more accurate analysis in qPCR. The lowest concentration with 95% probability of detection (confidence interval) defined by Napierian logarithm on probit analysis was 1.13 copies for SYBR™ Green and 1.17 copies for TaqMan™.

LoD in conventional PCR with primers Pv1/Pv5 was 10^-1^ p/µL and 1 p/µL with Pv1/ Pv6, while real-time PCR for both SYBR™ Green and TaqMan™ systems showed the same result (10^-2^ p/µL).

*Analytical specificity* - The other species of human malaria tested (*P. falciparum*, *P. malariae*, *and P. ovale*) as well as negative controls and NTC were not amplified in both SYBR™ Green and TaqMan™ systems.

*Precision* - The analysis of precision within triplicates and among nine runs showed 100% agreement at 100, 10, and 1 p/µL, for real-time and conventional PCRs. However, the SYBR™ Green system also reached 100% of agreement within and between runs for the lower concentration (0.1 p/µL). Below 0.1 p/µL, the results showed a random agreement pattern across the different concentrations and samples tested (Supplementary data II).

Considering the reproducibility between runs using two different operators, again the TaqMan™ system for real-time, and conventional PCRs maintained 100% reproducibility until 1 p/µL, while for SYBR™ Green agreement was maintained until 0.1 p/µL (Supplementary data II).

**TABLE I:** TaqMan™ single and multiplex reactions with 0.12 e 120 parasites/µL

Parasitaemia p/μL	Reaction	*Plasmodium vivax*		*RNAseP*	
		Ct mean	Ct SD	Ct mean	Ct SD
120	M	29.4	0.2	24.3	0.1
120	S	29.8	0	--	--
120	S	--	--	24.2	0
0,12	M	37.1	0	24.8	0
0,12	S	36.0	0	--	--
0,12	S	--	--	24.7	0.2

p/ µL: parasites per microliter; M: multiplex; S: singleplex; Ct: threshold cycle; SD: standard deviation.

For the two GeneAmp™ PCR thermal cyclers, 100% agreement in repeatability and reproducibility (100 to 1 p/µL) was observed. Conversely, Veriti™ Fast with both primer sets (Pv1/Pv6 and Pv1/Pv5) did not exhibit 100% agreement even at the higher concentrations, generating mean κ values indicative of good (0.76) for Pv1/Pv6, or moderate (0.540) agreement for Pv1/Pv5 (Table II).

*Discussion* - The guidelines outlined by the WHO to eliminate *P. vivax* highlights important challenges, such as the detection of subclinical cases or “asymptomatic reservoirs”.^4^ ^,^ ^20^ Frequently, such asymptomatic infections are missed when using routine microscopy diagnosis, thus contributing to transmission maintenance.^21^ ^,^ ^22^ Since the advent of PCR methodology, it has improved in sensitivity and performance as compared with microscopy, and has advanced information and resources for malaria control programs.^23^ However, performance of molecular diagnostics needs to be addressed by robust validation of tests under ideal conditions, such as those of reference centres.^6^ ^,^ ^17^ ^,^ ^24^ Since our laboratory is a reference laboratory for malaria diagnosis for the Brazilian Ministry of Health, evaluations of reliability (precision) and sensitivity of *in-house* PCRs for detection of low parasitaemic *P. vivax* individuals, in real-time and conventional assays, were performed.

The *in-house* conventional PCR used in routine diagnosis has showed satisfactory detection of low parasitaemia, but rapidity for obtaining results is required for emergency cases. Real-time PCR can reduce the turnaround time of conventional PCR by at least 2 h and allows the quantification of parasites for follow-up treatment. Consequently, a real-time PCR assay was developed and validated.

The lack of reproducibility between conventional and real-time PCR has been reported.^11^ ^,^ ^25^ Notwithstanding, the comparison between different PCR protocols can be hindered due to different amplification targets, reagents, and standardisation procedures, and, as a result, it is not clear how sensitive and reliable the tests are.^9^ ^,^ ^24^

To avoid these concerns, both PCR formats were carried out using the same primers, samples, and experimental design. In addition the standardisation of conventional and real-time PCRs was performed in accordance with the Food and Drug Administration (FDA) and Minimum Information for Publication of Quantitative Real-Time PCR Experiments (MIQE) guidelines to ensure the reliability required to achieve high-quality data.^17^ ^,^ ^18^

Since it is well-known that quantitative efficiency does not apply to qualitative tests, this parameter was only assessed in real-time assays using SYBR Green or TaqMan™ systems through a cloned cysteine proteinase *P. vivax* DNA standard curve. In this way, a slope close to 100% generated a linear range that was directly proportional to the analyte concentration in the samples where the lowest concentration corresponds to the LoQ. As a result, the reportable linearity range rose up to 10^2^ DNA copies for both SYBR™ Green and TaqMan™ systems. Nonetheless, the SYBR™ Green system showed a large dispersion of Ct values, generating high standard deviations, leading us to conclude that when parasite quantification is need, TaqMan™ should be used due to its lower standard deviation.

Considering that the LoD is of great interest to determine treatment endpoints, the lowest number of p/μL that could be detected in PCR assays was determined. In this respect, it must be considered that, in microscopic examination, the parasitaemia counts do not necessarily reflect genome numbers in clinical samples (one schizont can harbour at least 12 merozoites/12 DNA copies). In view of this restriction, a cloned *P. vivax* cysteine proteinase gene was employed to calculate the real-time PCR LoD in SYBR™ Green singleplex and TaqMan™ multiplex systems. The LoD of TaqMan™ (1.17 copies/µl) was comparable to SYBR™ Green (1.13 copies/µl), demonstrating that the less expensive SYBR™ Green could replace TaqMan™ system when parasite quantification is not required.

**TABLE II:** A synopsis of performance characteristics of real-time and conventional polymerase chain reaction

Performance characteristics	Real-time PCR		Conventional PCR	
	SYBR™ Green	TaqMan™	Pv1/Pv5	Pv1/Pv6
Efficiency (%)	104.2	98.9	NA	NA
Coefficient correlation (r^2^)	0.99	0.98	NA	NA
LoQ (copies/ µL)	10^2^	10^2^	NA	NA
Analytical sensitivity (LoD) (copies/µl or parasites/µL)	1.13	1.17	10^-1^	1
Repeatability (within run)^*a*^	0.1	1	1	1
Reproducibility^*a*^ (between run)	0.1	1	1	1
Reproducibility^*a*^ (between operators)	0.1	1	1	1

*a*: smallest parasitaemia with 100% of agreement results; NA: not appropriated; p/µL: parasites per microliter.

Concerning the LoD of conventional PCR, the reverse primer Pv5 displayed better performance (0.1 p/µL) compared to Pv6 (1 p/µL). The increased sensitivity with the Pv5 primer indicates that, even using the same DNA target, amplification efficiency is related to primer characteristics, which can decrease primer-dimers and reduce undesirable clamp formation, thereby improving the sensitivity. Comparing real-time and conventional PCRs using the Pv5 primer, the LoD was increased to 0.01 p/µL, suggesting that real-time PCR is more sensitive than conventional PCR. The LoDs previously reported in real-time PCR using TaqMan™ varied from 1.5 (25), 1.13,^10^ 0.2,^24^ to 0.02 p/µL,^26^ using SYBR Green™ from 1^27^ to 2 p/µL,^28^ and in conventional nested-PCR LoDs ranged from 5 to 2 p/µL.^28^ These data lead us to conclude that the conventional and real-time PCRs developed presented higher sensitivities. The LoD value is also important to determine the concentration to be used as a low positive control, which should be monitored to ensure consistency of performance between runs at levels near the cut-off, and to ensure that the LoD does not change when new reagent lots are used.^17^

Reliability is another important measure of data quality in PCR. The term precision refers to how a given measurement can be reproduced when it is repeatedly applied in a test, using multiple aliquots of a single sample under same conditions, according to FDA guidelines.^17^ ^,^ ^18^

Thus, to guarantee that precision was not a limiting factor of success in *P. vivax* diagnosis, reproducibility (between runs, operators, and instruments) and repeatability (within-run) were investigated. Interestingly, 100% of agreement within and between runs was verified up to 1 p/µL in both real-time and conventional PCRs, regardless of the primer set. In real-time assays, the SYBR™ Green system was more reliable, since 100% of agreement was also displayed in dilutions corresponding to 0.1 p/µL. It could be argued that SYBR™ Green is a nonspecific dye and, consequently, primer-dimers may generate spurious products (or artefacts), causing false positive parasite detection; however, the SYBR™ Green melt curve analysis validated the specificity of SYBR™ Green assays.

Curiously, although it was possible to detect as low as 0.0000001 p/µL, below 0.1 p/µL the agreement occurred by chance, without a direct relationship with parasite numbers (Supplementary data II). This result was somewhat expected because loss of reproducibility under submicroscopic conditions when the initial molecule number is very low together with small sample volumes (2 µL) has been reported.^29^ In fact, lack of reproducibility and repeatability is due to the variation errors resulting from the stochastic distribution of the target DNA molecule, generating the so-called Monte Carlo effect.^29^ ^,^ ^30^ The same is true for the variation between operators, reflecting the probability of a determined number of molecules that are present in an aliquot pipetted from a solution with a very small number of DNA target copies, rendering the random variation by sampling error (Poisson’s law) significant.^29^ ^,^ ^30^ In view of these, performing tests in triplicate is strongly recommended to diagnosis asymptomatic infections to minimise diagnostic errors when submicroscopic parasitaemia is present.

Taking into account that reference laboratories may have different models of thermal cyclers, concordance between two conventional PCR instruments, GeneAmp™ and Veriti™ fast, was tested using the same reagents. In this way, Veriti™ fast showed a loss of reproducibility, independently of the primer set and sample concentration used. This outcome could be a result of the Veriti™ fast characteristics, due to the higher speed in temperature ramp during each cycle, likely leading to false negative results in the experiments reported here.

With respect to the analytical sensitivity (LoD) and precision of both conventional and real-time PCRs in SYBR™ Green and TaqMan™ systems, no expressive difference was observed between these PCRs formats. The choice for PCR format should consider the diagnosis urgency, need for parasitaemia quantification, and cost. When faster results or parasitaemia quantification for follow-up disease evolution are needed, the real-time TaqMan™ system PCR is the most appropriate choice.

In summary, the precision and sensitivity of conventional and real-time PCRs were set up and fully validated for *P. vivax* detection in a reference diagnostic centre. To diagnose *Plasmodium*-infected-asymptomatic individuals, in which most infections are subpatent parasitaemia, it is recommended that tests be performed in triplicate to minimise diagnostic errors.
